# Starting insulin in type 2 diabetes: Overcoming barriers to insulin therapy

**DOI:** 10.4103/0973-3930.43102

**Published:** 2008

**Authors:** Udaya M. Kabadi

**Affiliations:** Department of Veterans Affairs, VAMC, Iowa City, IA and Roy and Lucille Carver College of Medicine, University of Iowa, Iowa City, IA, USA

**Keywords:** Diabetes mellitus type 2, treatment, insulin, oral hypoglycemic agents, dosage and schedules

## Abstract

**BACKGROUND:**

Several barriers to insulin therapy are encountered by both the providers and the patients with type 2 diabetes mellitus. These barriers include the fear of the needles i.e. number of injections as well as number of times of self blood glucose monitoring, fear of hypoglycemia and weight gain as well as the convenience, compliance and the cost. However, most of these patients are likely to require insulin therapy with increasing duration of the disorder because of the progressive cell failure. Therefore the most important aspect of insulin therapy must revolve around the regimen most suitable and acceptable because of its ability in overcoming these barriers while being effective in attaining and maintaining desirable glycemic control.

**METHODS:**

Recently published studies using different regimens with combinations of various oral agents and insulins in patients with type 2 DM and manifesting lapse of glycemic control when treated with various oral agents are discussed. Specific attention is paid to the capacity of each individual regimen in overcoming aforementioned barriers.

**RESULTS:**

Comparative analysis amongst various insulin regimens shows that combination of metformin, and glimeperide with SC administration of basal insulin Lantus required the least daily dose of insulin with least consequential hypoglycemia as well as weight gain. Moreover, the number of injections as well as the number of times of self blood glucose monitoring, were lesser with this regimen with better compliance and more convenience in comparison to other combination insulin regimens.

**CONCLUSION:**

The insulin regimen with fewest barriers consists of one SC injection of basal insulin lantus in combination with oral agents. However, to be effective, oral agents must include a secretogogue i.e. glimeperide in addition to a sensitizer i.e. metformin and not multiple sensitizers without a secretogogue. Moreover, this regimen apparently is also the most preferred by the patients, and is cost effective.

Both health care providers and patients perceive insulin therapy in type 2 diabetes mellitus (DM) as being associated with several barriers [Table 1]. As a result, insulin therapy is frequently, and unfortunately, delayed and it is often deemed to be an option of the last resort. Yet another reason for the delay is the misconception that type 2 DM is a disorder caused by insulin resistance alone and therefore insulin administration is irrational. However, two studies, including UKPDS, have clearly demonstrated a decline in pancreatic β-cell function at the time of diagnosis.[1,2] In fact, higher the fasting plasma glucose, greater is the reduction in β-cell function at the time of diagnosis.[1,2] Moreover, a recent study has documented that the decline in the postprandial first-phase insulin secretion occurs simultaneously with worsening insulin resistance.[3] Both impaired glucose tolerance and postprandial hyperglycemia are caused by progressive decline in first-phase insulin secretion. By the time the disease progresses to fasting hyperglycemia, progressive fall in the second-phase insulin secretion is also documented.[4–6] Thus, even at the time of diagnosis, type-2 DM is characterized by a dual defect, namely, insulin resistance and deficient insulin secretion. Moreover, β-cell dysfunction is apparently progressive and as noted in UKPDS, there is a simultaneous worsening of insulin resistance that is caused by weight gain during the course of the disorder.[1] Therefore, insulin therapy is likely to be required in almost all subjects with type 2 DM if they survive long enough, because oral agents will then be unable to maintain desirable glycemic control. Insulin therapy is then used as an adjunctive modality or is substituted for oral agents.[6–8] The addition of insulin to the regimen, while actually reducing the cost of treatment, aids in attaining and maintaining glycemic control, which is well documented to delay the onset or retard the progression of complications.[9–11] Moreover, insulin is the only effective and approved therapy in certain situations and special populations [Table 2].

**Table 1:** Overcoming barriers to insulin therapy.

Fear of hypoglycemia Fear of weight gain and the consequences Fear of needles Fear of impending doom? Fear of atherogenesis (provider)? Inconvenience Number of injections Frequency of SMBG Quality of life Noncompliance • Cost

**Table 2:** Indications for insulin therapy in patients with type 2 diabetes.

Maximal doses of oral agents, even in combination, do not control glucose levels Presence of ketonuria, weight loss, and/or severe hyperglycemic symptoms Intercurrent illness Surgery, trauma, infection, or fever Pregnancy < 10 years of age*

* Metformin is approved for subjects with type 2 DM over the age of 10 years.

However, the type of insulin regimen to be used in subjects who manifest lapse of glycemic control while receiving oral agents is still debated. These regimens have included combinations of different oral agents and various insulin formulations, including analogs. Several clinical trials comparing different combination regimens have been published. However, these studies have yielded contradictory results, and all of them have tended to focus more on efficacy, paying less attention to the barriers to insulin therapy [Table 1].

In two studies,[12,13] twice-daily subcutaneous (SC) insulin analogs [ie, 75/25 Humalog Mix (Eli Lilly Inc.) or 70/30 Novolog Mix (Novo Nordisc Pharma)] provided greater glycemic control than adjunctive therapy with once-daily SC basal insulin glargine (Sanofi-Aventis Pharmaceuticals) when used in combination with either metformin alone of with metformin and pioglitazone. However, both the number of hypoglycemic events and the amount of weight gain were greater with twice-daily premixed insulin than with once-daily insulin glargine, which could possibly be attributed to the significantly higher daily insulin dose with the former regimen.

In contrast, in another study,[8] once-daily (morning) administration of insulin glargine in combination with glimepiride and metformin was more effective in controlling glycemia than insulin monotherapy consisting of twice-daily SC administration of premixed 70N/30R insulin. Moreover, the number of hypoglycemic events and the weight gain were markedly lower in the subjects receiving combinations of insulin glargine with glimepiride and metformin than in those receiving twice-daily premixed 70/30 insulin monotherapy. This could be attributed to the markedly lower daily dose of insulin glargine (28 units) in comparison to that of premixed 70/30 insulin (65 units). Finally, recent studies using insulin glargine in the morning in combination with oral agents (eg, glimepiride) showed marked improvement in glycemic control and no weight gain.[14,15]

Another recent study using combinations of once-daily insulin glargine with glimepiride alone showed a marked improvement in glycemic control with an acceptable rate of hypoglycemia and minimal weight gain.[16] Moreover, we have demonstrated that the daily insulin dose required to attain and maintain a desirable glycemic goal (HbA1C ≤ 7.0) was the least (0.21 units/kg body weight) when insulin was used in combination with both glimepiride and metformin as compared to insulin used in combination with any these individually or when used with placebo[17] and consequently, the number of hypoglycemic events and the weight gain were lowest with this combination. Finally, a recent comparison between insulin glargine and insulin detemir given along with oral agents showed that both yielded similar improvements in glycemic control.[18] However, insulin detemir was required to be used twice daily in a majority of patients in contrast to insulin glargine, which needed to be administered only once a day in all patients. The number of hypoglycemic events and the weight gain were similar in both groups; however, compared to the dose of insulin glargine (0.4 U/kg/day), the daily dose of insulin detemir was double (0.8 U/kg /day) in patients using it twice daily and 1.5 times (0.6 U/kg /day) in those using it once a day. The requirement for twice-daily administration of insulin detemir in most patients may be attributed to lack of its peakless basal profile as well to its duration of action being less than 24 h. Finally, we believe that the weight gain noted with insulin glargine in this study could have been prevented if it had been administered in the morning as was done in other studies.[14,15]

Thus, it is apparent that various insulin regimens, including insulin monotherapy, are effective for attaining glycemic control, but the rates of adverse outcomes such as hypoglycemia and weight gain vary with the different regimens. It is also apparent that the choice of oral agents is crucial when using combination regimens. It is difficult to fathom the reason for the extremely high daily insulin dose required to improve glycemic control when it is used in combination with either metformin or/and pioglitazone[12] since these agents are deemed insulin sensitizers. However, this high daily dose is consistent with previous reports on the use of a combination of insulin with either pioglitazone or rosiglitazone.[19,20] It is possible that these agents fail to improve sensitivity to exogenous insulin. Furthermore, the absence of an insulin secretagogue (eg, glimepiride) in the regimens tested may have contributed to the lower efficacy of basal insulin glargine and hence the need for injections of rapid-acting insulin prior to meals to control postprandial glycemia. This inference is further confirmed by other studies in which adjunctive therapy consisting of insulin glargine with glimepiride alone or in conjunction with metformin were very effective in improving overall diurnal glycemic patterns including preprandial and postprandial glycemia.[8,16] Finally, the importance of including an insulin secretagogue, ie, glimepiride, in the oral drug combination is further evident in a study in which just a once-daily presupper SC administration of Novomix 70/30 insulin was adequate for achieving desirable glycemic control[17] in contrast to the requirement for the use of twice-daily SC injection of the same insulin when used with insulin sensitizers.[12,13] We believe that basal insulin administration blunts hepatic glucose production with the help of metformin and improves fasting glycemia. Simultaneously, basal insulin also inhibits release of insulin, promoting insulin storage in the β cells. Insulin secretagogues, like glimepiride, promote the release of stored insulin in response to meals and therefore ameliorate postprandial hyperglycemia.[4,5] None of the insulin sensitizers demonstrate this insulin-releasing property. Therefore, as noted previously, twice-daily administration of both long-acting insulin and rapid-acting insulin is necessary to control preprandial and postprandial glycemia when used in combination with metformin or/and a glitazone.[12,13] In contrast, if glimepiride and metformin are used, once-daily administration of basal insulin is sufficient for improving glycemic control.

Finally, it is clear from these studies, that the greater the daily insulin dose the greater the number of hypoglycemic events and the greater is the weight gain and, probably, its long-term consequences. The dropout rates of subjects using twice-daily premixed insulin in these studies were significantly higher than that of subjects receiving once-daily insulin glargine.[8,12] This finding may be because of the fear of needles as well as the inconvenience caused by more frequent injections and the need for more frequent SBGM. In contrast, a regimen consisting of basal insulin in combination with appropriate oral agents causes the least hypoglycemia and weight gain and requires the least daily insulin dose, with the fewest injections and minimum SMBG and is likely to provide the maximum convenience, the highest compliance, and the best quality of life in the most cost-effective manner.[21] To conclude, we believe that the best therapeutic approach in subjects manifesting lapse of glycemic control while receiving oral agents is an adjunctive therapy consisting of basal insulin glargine in combination with both a secretagogue like glimepiride and a sensitizer like metformin in the absence of contraindications for these agents.
